# Supplementary figures and images for: The Autotrophic Core: An Ancient Network of 404 Reactions Converts H2, CO2, and NH3 into Amino Acids, Bases, and Cofactors
Source: Microorganisms. 2021 Feb 23;9(2):458. doi: 10.3390/microorganisms9020458 (PMC7926472; doi:10.3390/microorganisms9020458)

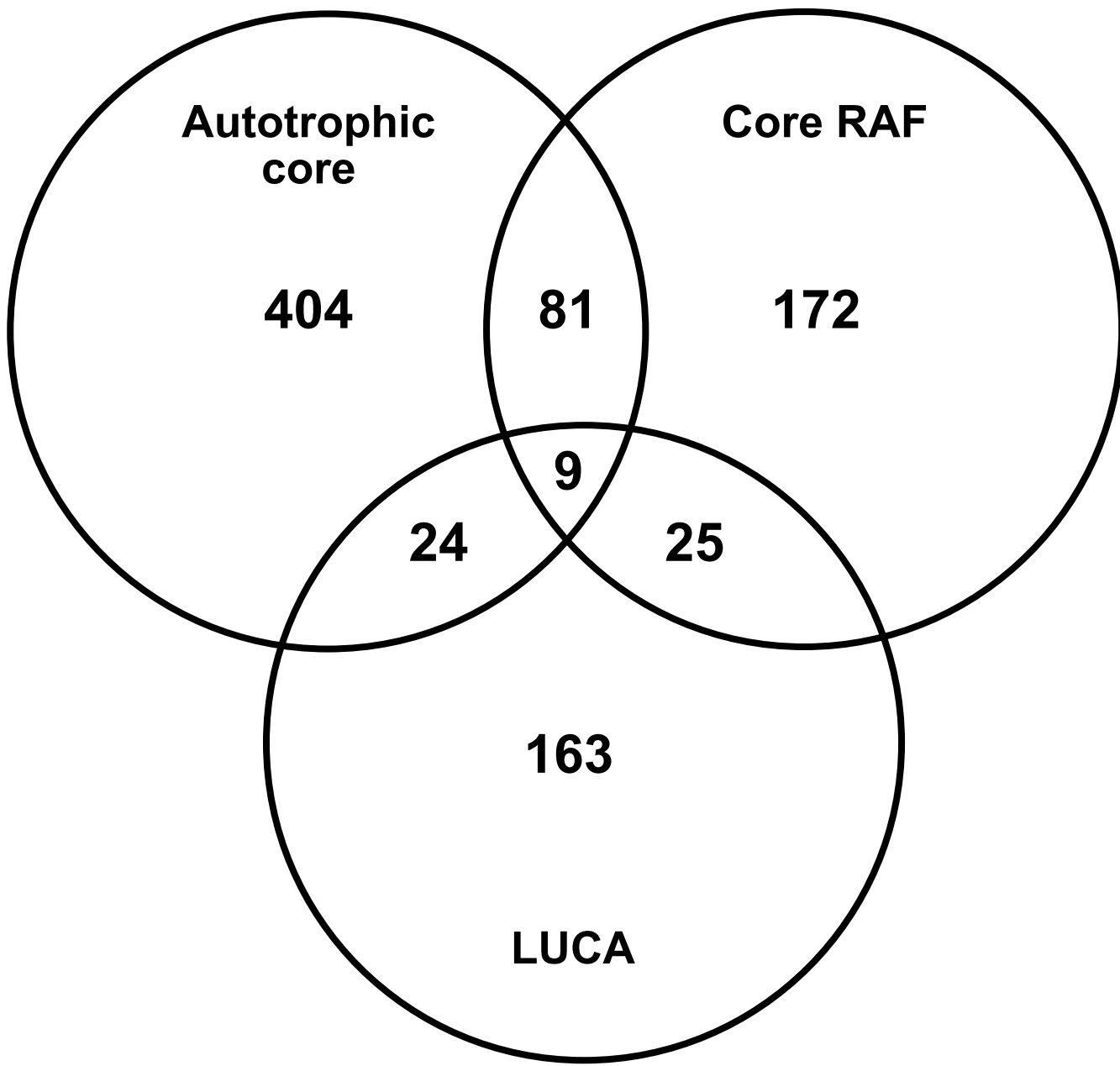

Supplement: Supplementary file 1 [file microorganisms-09-00458-s001.zip › Supplementary_Figure_1.pdf]
